# Hands-On Versus Hands-Off Treatment of Hip-Related Nonspecific Musculoskeletal Diseases: A Systematic Review

**DOI:** 10.3390/jfmk9040262

**Published:** 2024-12-07

**Authors:** Giulia Franceschi, Irene Scotto, Filippo Maselli, Firas Mourad, Marco Gallotti

**Affiliations:** 1 Department of Human Neurosciences, Sapienza University of Rome, 00185 Rome, Italy; giuliafranceschi98@gmail.com (G.F.); irenescotto.107@gmail.com (I.S.); masellifilippo76@gmail.com (F.M.); 2 Department of Physiotherapy, LUNEX International University of Health, Exercise and Sports, 4671 Luxembourg, Luxembourg; firas.mourad@lunex-university.net; 3 Luxembourg Health & Sport Sciences Research Institute A.s.b.l., 50, Avenue du Parc des Sports, 4671 Luxembourg, Luxembourg

**Keywords:** hip disease, hands-on, hands-off, manual therapy, exercise therapy, effectiveness

## Abstract

**Background/Objectives:** A manual approach combined with therapeutic exercise versus therapeutic exercise alone is a debated issue in the literature. The American College of Rheumatology guidelines “conditionally recommended against” manual therapy for the management of hip osteoarthritis. Manual therapy followed by exercise, instead, appears to lead to a faster return to sport than exercise alone for adductor groin pain. There is a need to understand which is the most effective treatment in the management of hip nonspecific musculoskeletal diseases. The aim of this systematic review is to determine which is the most effective treatment between manual therapy combined with therapeutic exercise and therapeutic exercise alone in subjects with hip nonspecific musculoskeletal diseases. **Methods:** This systematic review complies with the guidelines of the 2020 Prisma Statement. The databases consulted were Pubmed, Cinahl, and Web Of Science. The search was conducted from October 2004 to November 2023. The search string was developed following the PICO model. Free terms or synonyms (e.g., manual therapy, exercise therapy, hip disease, effectiveness) and Medical Subject Headings terms were combined with Boolean operators (AND, OR, NOT). The risk-of-bias assessment was conducted using Version 2 of the Cochrane risk-of-bias tool for randomized controlled trials and the Newcastle Ottawa Scale for observational studies. A qualitative analysis of the results was conducted through narrative synthesis of key concepts. When possible, quantitative analysis was conducted through statistical parameters. **Results:** Ten articles were analyzed. Results show no differences between the interventions analyzed. Preliminary evidence seems to favor the combined intervention for the outcomes of pain, ROM, and patient satisfaction, with other studies claiming an absence of differences. Only one study claims that therapeutic exercise alone is more effective for quality of life. Preliminary evidence seems to show that manual therapy does not seem to bring any benefit in addition to therapeutic exercise in mid- and long-term functionality, especially for hip osteoarthritis. **Conclusions:** There seems to be no difference in effectiveness between manual therapy combined with therapeutic exercise and therapeutic exercise alone in individuals with hip nonspecific musculoskeletal diseases.

## 1. Introduction

The choice between a manual approach (“hands-on”) combined with therapeutic exercise and therapeutic exercise alone (“hands-off”) is an extremely debated issue in the musculoskeletal literature in different districts [1,2,3,4,5,6,7,8,9,10,11,12]. Regarding musculoskeletal disorders of the hip, there is conflicting evidence about the best treatment between manual therapy combined with therapeutic exercise and therapeutic exercise alone [2,3,4,5,6,7,8,9]. For the management of hip osteoarthritis, the 2019 OARSI guidelines mainly recommend therapeutic exercise and education (level 1A), followed by mind–body exercise (tai chi), NSAIDs, walking aids, Cognitive Behavioral Therapy, manual therapy, weight management, and good clinical practice statements (level 1B) [13]. The American College of Rheumatology guidelines advise against (“conditionally recommended against”) manual therapy combined or not with therapeutic exercise, probably because the desirable effects outweigh the undesirable effects [14]. However, Hoeksma’s study shows that manual therapy based on specific mobilizations and manipulations appears to be a better approach compared with a therapeutic exercise program [15].

This contradictory nature of today’s literature on the management (hands-on vs. hands-off) of musculoskeletal disorders of the hip is also reflected in other types of pathologies like the management of long-standing adductor-related groin pain [9]. Weir’s study shows that the approach based on manual therapy followed by stretching and a return-to-running program appears to lead to a significantly faster return to sport than therapeutic exercise alone. However, neither treatment proved to be particularly effective [9]. The best treatment of Great Trochanteric Pain Syndrome (GTPS) involves the combination of education, strengthening, and neuromuscular control exercises, but, despite the evidence, a percentage of physical therapists use additional therapies such as manual therapy, stretching, and massage without need [16]. Manual treatment of deep gluteal syndrome appears to be effective when based on myofascial treatment techniques, but it is unclear whether the combination of stretching exercises would lead to better results, given the low methodological validity of the existing studies [17]. The lack of clarity as to which strategies are most useful for the management of specific musculoskeletal conditions, given their increasing prevalence, could lead to a waste of time and resources in the management of this type of patient.

Therefore, the aim of this systematic review is to analyze the evidence on which is the most effective treatment between manual treatment combined with therapeutic exercise and treatment based on therapeutic exercise alone for the management of hip-related nonspecific musculoskeletal diseases. 

## 2. Methods and Materials

This systematic review was conducted following the update Preferred Reporting Items for Systematic Reviews and Meta-Analyses (PRISMA) 2020 Statement [18].

### 2.1. Protocol and Registration

As required by the guidelines for methodologically sound drafting of systematic reviews, the protocol of this systematic review was prospectively registered on the International Prospective Register of Systematic Reviews (PROSPERO) on 19 December 2023 (registration number: CRD42023481560) to avoid duplication of effort, reduce reporting bias, and promote transparency.

### 2.2. Eligibility Criteria

Studies, to be eligible, had to include adult subjects (age between 18 years and 65 years old) suffering from hip-related musculoskeletal disorders, such as femoroacetabular impingement (FAI), adductor-related groin pain, gluteal tendinopathy, hip osteoarthritis, GTPS, deep gluteal syndrome (DGS), snapping hip, trochanteric bursitis, or meralgia paresthetica. The experimental treatment of the included studies had to include manual therapy techniques, such as tractions, mobilizations in the translation of the joint, mobilizations with movement (MWM), manipulations (with or without thrust), soft specific tissue mobilization techniques (such as muscle energy techniques (MET), deep transverse massage, ischemic pressure) combined with therapeutic exercise, mainly consisting of mobility, strengthening, stretching, endurance, and motor control exercises. For comparison, instead, the studies had to include only therapeutic exercise in the modalities previously described.

The outcomes investigated by this systematic review were pain, investigated by patient-related outcome measures (PROMS) such as Visual Analogue Scale (VAS) and Numeric Pain Rating Scale (NPRS); range of motion (ROM); function, investigated by PROMS or functional performance testing; quality of life, investigated by PROMS; and patient satisfaction (investigated by PROMS or survey).

Studies, to be eligible, had to be only primary studies, such as randomized controlled trials (RCTs), or observational studies like cohort studies, case–control studies, case series, and case reports. The inclusion and exclusion criteria are shown in Table 1.

### 2.3. Literature Search

The literature search was conducted in November 2023, and results obtained range from October 2004 to November 2023 in PubMed, Cinahl, and Web of Science databases. A search string was generated according to the PICO model (patient, intervention, comparison, and outcome) to assess the quality and comprehensibility of the literature search. The search was conducted by two blinded and independent reviewers (GF, MG), and any disagreements were resolved by consulting a third reviewer (IS). Search terms describing hip musculoskeletal disorders, manual therapy, therapeutic exercise, and outcomes of interest were combined to create a search string with appropriate words (keywords). The search strategy for Medline was developed using the PICO strategy. Keywords such as free terms and synonyms and, when possible, MeSH (Medical Subject Headings) terms were combined with the Boolean operators AND, OR, and NOT. Table 2 summarizes the example of the search terms used for Medline.

### 2.4. Study Selection

After eliminating duplicates using Rayyan software (https://www.rayyan.ai/ accessed on 4 April 2024) [19], two reviewers (GF, MG) independently screened the studies by title and abstract. If discrepancies arose, they were solved by consulting between authors; if the conflict was persistent, then a third reviewer (IS) was consulted to solve the conflict. Subsequently, the eligibility of the full texts was assessed, and reasons in case of exclusions were recorded. 

### 2.5. Data Extraction

The four reviewers (GF, MG, IS, FMa) independently extracted and included data from the included studies into a standardized Excel spreadsheet containing title; type of study; authors; year and journal of publication; total number of patients (divided by sex and age); type of musculoskeletal disorder and time of onset; description of experimental group intervention; description of intervention of the various control groups; primary and secondary outcomes; follow-up and evaluation time points; description of results; any secondary analyses; and notes. In case of discrepancies, a fifth author (FMo) was consulted to solve conflicts. Data were extracted in parallel by two authors (GF and MG) to reduce the risk of bias [20].

### 2.6. Data Synthesis

The outcomes analyzed in this review are both qualitative and quantitative. Once data extraction was completed, a single reviewer (GF) grouped all studies focused on a specific outcome of interest and summarized the results by presenting the key concepts and the most shared elements between the studies through a textual description. This process was considered due to the heterogeneity of the included studies. Furthermore, when possible, quantitative analysis was conducted through different approaches depending on the outcome of interest. Statistical tools such as intervals, mean, and differences between means were used. Tables and graphs were used to summarize the data for visual explanation.

### 2.7. Assessment of Risk of Bias

Risk of bias (RoB) was independently assessed by two reviewers (GF, MG). The RoB version 2 tool [21] was used to assess randomized controlled trials (RCTs) to consider all potential biases, including the randomization process, deviations from intended interventions, missing outcomes, outcome measures, and selection of reported outcomes. In addition, an overall risk-of-bias judgment was determined using the RoB 2 tool. Two reviewers (GF and MG) examined each domain of the RCTs and judged the overall risk of bias as “low”, “some concerns”, or “high”. The NOS scale for nonrandomized studies was used for retrospective case–control studies, observational studies, and prospective observational cohort studies [22]. The scale includes 9 items investigating 3 domains: (i) sample selection (4 items), (ii) comparability (2 items), and (iii) outcome (3 items) for case–control and cohort studies, respectively. A limit for methodological quality has not yet been validated for observational studies using this scale [22]. However, although the NOS does not allow for a quantitative score, each star attributable to a single NOS element could be considered as a point, with scores ranging from 0 to 9 for the NOS [22].

## 3. Results

**Figure 1:**
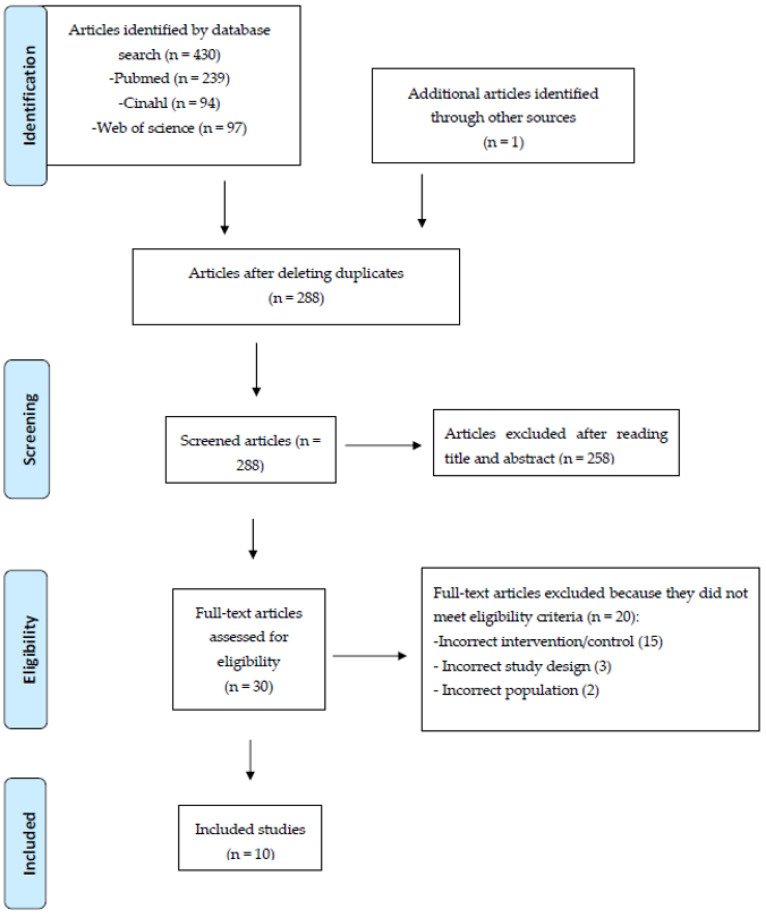
PRISMA statement flow chart.

The process of study selection and article inclusion is shown in the PRISMA Statement flowchart in Figure 1. 

A total of 431 articles were originally identified. In total, 143 of these were eliminated as duplicates. The remaining 288 articles were analyzed by title and abstract. A total of 258 of these were eliminated because they did not meet the inclusion criteria. The remaining 30 articles were analyzed by full-text, and 10 were included in the systematic review. The 10 included studies were published from 2004 to 2023 [2,3,4,5,6,7,8,9,15,23]. All 10 included studies are randomized controlled trials (RCTs), and 3 out of these 10 are pilot studies [4,5,6]. In total, 2 studies represent secondary analyses (long-term follow-ups) of studies already included in these 10 and are described as a single study [3,23]. The characteristics of the included studies can be further detailed in Supplementary File S1.

### 3.1. Population 

A total of 597 patients were included. Population characteristics are summarized in Table 3. In Abbott’s study [2], 113 of 206 patients suffered from knee osteoarthritis, so they were not considered. In Hoeksma’s study [15], osteoarthritis’ time of onset was not specified in the analyzed subjects. In Mahmoud’s study [7], paresthetica meralgia’s time of onset was not reported.

Most patients underwent manual therapy and therapeutic exercise interventions. Only two studies included the additional administration of “usual care” (unspecified dosage of analgesic drugs) by a general practitioner [2,4]. The type of intervention administered in detail is reported in Table 4.

### 3.2. Intervention 

Seven studies [2,4,5,6,7,8,9] out of eight evaluated the comparison between manual therapy combined with therapeutic exercise and therapeutic exercise alone. Only Hoeksma’s study [15] evaluated the comparison between manual therapy alone and therapeutic exercise alone. In particular, the manual therapy group underwent physiotherapist-mediated stretching techniques of the identified shortened hip muscles, followed by hip traction and manipulations in each restricted position [15]. In this study, the therapeutic exercise group performed resistance exercises (treadmill/cyclette), strength exercises (using weights and specific equipment), and coordination exercises (gait with increasing complexity and balance exercises). This was followed by exercises to increase ROM (stretching, passive hip movements, 3D active movements in unloading and loading in standing, sitting, and lying positions), exercises to reduce pain (stretching, active exercises), and walking exercises (gait pattern training and possible use of walking aids, stair training) [15].

In Harris-Hayes’ study [6], the combined group underwent impairment-based joint mobilization techniques. For example, if hip flexion was limited, the manual techniques administered were passive flexion, caudal glide, anteroposterior (hereinafter AP) glide, mobilization with movement, a combination of flexion, adduction, and internal rotation. This was followed by a home exercise program that included joint movement exercises and commonly used stretching to supplement the techniques administered during the sessions. The therapeutic exercise group performed exercises that included repeated practice of movements chosen according to the patient’s functional demands using optimized movement patterns. Depending on the patient’s performance, the difficulty of specific tasks was increased by increasing repetitions, load, or changing the supporting surface. For example, if the task was squatting, the training included various levels of exercise: slow lowering into the chair without resistance; slow lowering with resistance; slow lowering with only touching the chair with resistance; and bilateral squatting without resistance and with resistance. Then, this group performed the same home exercise program as the manual therapy group [6].

In Weir’s study [9], the combined group underwent paraffin wraps on the adductor insertion, followed by operator-dependent stretching techniques of the same muscles. Following this treatment, a 5 min warm-up (slow jogging/cycling), bilateral adductor stretches, and finally, a 10 min immersion in a hot bath were conducted. After 14 days, if there was no pain, the athlete began a return-to-running program. The therapeutic exercise group performed two exercise modules (the first one for two weeks, the second one starting from the third week). The first module mainly involved strengthening exercises of the adductor, hip flexors, abdominal muscles, and balance exercises. The second module mainly involved strengthening exercises of the abductor, hip flexor, and abdominal muscles, lumbar extension exercises, coordination and balance exercises, and sliding exercises on sliding surfaces. Then, the same return-to-running program as the combined group was undertaken [9].

In Wright’s study [5], the combined group underwent manual therapy technique (manipulative technique with or without thrusts) and exercise (targeting muscle strengthening, stretching, and motor control). Physical therapists used impairment-based treatment to minimize variation. This group then performed an exercise program at home that included 6 exercises targeting hip strength and flexibility. The therapeutic exercise group performed the same home exercise program as the combined group [5]. 

In French’s study [8], the combined group underwent general mobilization followed by 2–5 treatment techniques chosen according to the two most restricted (impairment-based approach) movements (e.g., if symptoms were related to hip flexion, supine AP and mobilizations with movement in flexion with a belt were performed; if symptoms were related to extension, instead, prone posteroanterior (hereinafter PA) glide, lateral PA, and hip flexor muscle stretching were performed). An exercise protocol was then performed, including a warm-up phase (5 min of cyclette), a stretching phase (stretching of the muscles that most limited two movements), and a strengthening phase targeting the gluteal muscles (low-load exercises starting from unloaded positions and progressing to more loaded positions). The therapeutic exercise group performed the same exercise protocol as the combined group, supplemented by a home exercise program that included up to five exercises, including two stretching postures. This study also included an additional control group on the waiting list for physical therapy to evaluate the possible impact of the wait-and-see approach on symptoms [8]. 

In Abbott’s study [2], the manual therapy group underwent primary interventions (mandatory) and secondary interventions (nonmandatory, prescribed if indicated by the physical therapist evaluation). The primary ones included axial and lateral hip traction techniques, PA, AP, stretching techniques, and soft specific tissue mobilization techniques; the secondary ones included knee flexion and extension, proximal tibio-peroneal joint manipulation, patellar gliding, ankle traction, ankle plantar soft tissue treatment, and lumbopelvic manipulation. This intervention was followed by a program of 6 mobility exercises at home. The therapeutic exercise group performed a supervised multimodal program of primary interventions (mandatory) and secondary interventions (nonmandatory, prescribed if indicated by physical therapist evaluation). The primary ones included aerobic exercise (10 min of walking/biking), muscle strengthening (hip abduction–extension–lateral rotation, knee extension), stretching (hip flexors, knee extensors, hip extensors, knee flexors hip abductors, and lateral hip rotators, ankle plantar flexors), and neuromuscular control exercises (weight shifting exercises while standing, balancing on uneven surfaces, lateral stepping/forward–backward/stairs walking exercises). Secondary ones involved strengthening the trunk, ankle plantar flexors, and hip flexors muscles. This was followed by a program of 6 exercises at home. The combined group performed a combination of the manual therapy and the therapeutic exercise groups’ treatments. The usual care group did not receive any treatment other than the pharmacological one offered by the general practitioner [2].

In Blackman’s study [4], the combined group underwent stretching in hip flexion, abduction, extension, and medial rotation position, followed by self-stretching exercises in hip flexion, abduction, extension, and medial rotation position to maintain the obtained results. A program of 5 strengthening exercises was then performed at home, including sit-to-stand, mini squat, active standing hip extension and abduction, and supine bridge. The therapeutic exercise group performed the same strengthening program at home as the combined group [4].

In Mahmoud’s study [7], the combined group underwent two Muscle Energy Technique (MET) techniques, particularly the physiotherapist performed Post Induced Relaxation techniques (PIR) in hip extension and adduction position. Then, subjects were given TENS to the anterior thigh and flexibility exercises for the hip flexors (not specified in the article). The therapeutic exercise group also received TENS to the anterior thigh and flexibility exercises for the hip flexors (not specified in the article) [7].

### 3.3. Outcome

#### 3.3.1. Pain

All eight studies [2,4,5,6,7,8,9,15] evaluated the effectiveness of manual therapy combined with therapeutic exercise compared with therapeutic exercise alone on pain. For the evaluation of this outcome, four studies [4,5,9,15] used the Visual Analogue Scale (VAS), three studies [2,7,8] used the Numeric Pain Rating Scale (NPRS), and one study [6] used the Hip Disability and Osteoarthritis Outcome Score (HOOS) subscale and the Pain Pressure Threshold (PPT).

In Hoeksma’s study [15], a statistically significant effect of the manual therapy group compared with the therapeutic exercise group on pain at rest and during walking was found:

VAS scores for pain at rest at the 5-week follow-up improved by 5.4 mm in the manual therapy group, while they worsened by 3.7 mm in the therapeutic exercise group. At 17 weeks, they improved by 3.4 mm in the manual therapy group, while they worsened by 3.9 mm in the therapeutic exercise group. At 29 weeks, there were no statistically significant differences in either group. VAS pain scores during walking at the 5-week follow-up improved by 11.2 mm in the manual therapy group while improving by 1.7 mm in the therapeutic exercise group. At 17 weeks, they improved by 17.6 mm in the manual therapy group, while they improved by 5.1 mm in the therapeutic exercise group. At 29 weeks, they improved by 17 mm in the manual therapy group, while they improved by 4.5 mm in the therapeutic exercise group.

In Weir’s study [9], VAS pain scores at 0 and 16 weeks during sports activity improved significantly in both groups (22.8 mm in the combined group and 37.5 mm in the therapeutic exercise group), but the difference between the two groups was not significant. At the 6 and 24-week follow-ups, no statistically significant differences were found.

In Wright’s study [5], between-group differences for changes in VAS pain scores were not significant at the 7-week follow-up. However, both groups showed statistically significant improvements: the combined group improved an average of 17.6 mm, and the therapeutic exercise group improved an average of 18 mm.

In Blackman’s study [4], a statistically significant difference was found for change in VAS pain scores between the exercise group (mean = −4.45) and the combined group (mean = −18.20) at the 7-week follow-up. In this study, patients had a statistically significant difference in baseline pain scores, with the mean pain score being 62 in the combined group compared with a mean of only 39 in the therapeutic exercise group.

In French’s study [8], no significant difference was found between groups about pain evaluated through NPRS at the 9- and 18-week follow-ups.

In Abbott’s studies [2,3], there was no hip or knee split at the 1 and 2-year follow-ups; therefore, results could not be considered.

In Mahmoud’s study [7], NPRS pain scores improved by 5.53 points in the combined group while improving by 3.4 points in the therapeutic exercise group at the 4-week follow-up.

In Harris-Hayes’ study [6], both groups reported clinically important improvements in HOOS pain subscale scores at the 12-week follow-up: The combined group improved by 12.5 points, while the therapeutic exercise group improved by 10.9 points. After statistical evaluation, however, no statistically significant differences were found between the two groups. Regarding the Pain Pressure Threshold (PPT), no changes occurred. 

#### 3.3.2. Range of Motion

Five studies [4,7,8,9,15] evaluated the effectiveness of manual therapy combined with therapeutic exercise compared with therapeutic exercise alone regarding ROM.

In Hoeksma’s study [15], a statistically significant effect of manual therapy compared with therapeutic exercise on ROM was found: Flexion/extension at the 5-week follow-up improved by 14.5 degrees in the manual therapy group while improving by 1.3 degrees in the therapeutic exercise group. At 17 weeks, it improved by 15.2 degrees in the manual therapy group, while it improved by 4.4 degrees in the therapeutic exercise group. At 29 weeks, it improved by 13 degrees in the manual therapy group, while it improved by 4.5 degrees in the therapeutic exercise group. Internal/external rotation at the 5-week follow-up improved by 13.4 degrees in the manual therapy group while improving by 1.2 degrees in the therapeutic exercise group. At the 17 and 29-week follow-ups, there were no statistically significant differences in either group.

In Weir’s study [9], hip joint ROM did not change significantly after treatment in either group or between the two groups at the 6-, 16-, and 24-week follow-ups.

In French’s study [8], there were significant improvements in the treatment groups compared with the control group in degrees of ROM at 9 weeks: The therapeutic exercise group improved by 17.21 degrees, the combined group improved by 18.42 degrees, and the control group worsened by 1.15 degrees. No significant difference was found between the therapeutic exercise group and the combined group at the 9- and 18-week follow-ups. 

In Blackman’s study [4], there was a statistically significant difference in change in hip flexion at the 7-week follow-up between the therapeutic exercise group (mean = 5.00) and the combined group (mean = 13.10). No significant difference was found for hip internal rotation.

In Mahmoud’s study [7], a statistically significant difference was found for the change in ROM on the PKB test at the 4-week follow-up: the combined group improved by 49 degrees while the therapeutic exercise group improved by 28.04 degrees.

#### 3.3.3. Functionality

Six studies [2,4,5,6,8,15] evaluated the efficacy of manual therapy combined with therapeutic exercise compared with therapeutic exercise alone regarding functionality. For the evaluation of this outcome, six studies [2,4,5,6,8,15] used PROMS, and five studies [2,5,6,8,15] used functional performance tests. In particular, one study [15] used the Harris Hip Score (HHS) and the walking test; one study [6] used the Hip Disability and Osteoarthritis Outcome Score (HOOS) subscale, the International Hip Outcome Tool-33 (IHOT-33), and the evaluation of two-dimensional kinematic variables in the frontal plane (hip adduction, pelvic drop, trunk lean); two studies [4,5] used the Lower Extremity Functional Scale (LEFS), of which one study [5] also used the Hip Outcome Score (HOS) ADL and Sport subscale, the Single Assessment Numeric Evaluation (SANE) ADL, and Sport subscale and functional tests, such as deep squat, triple hop, hip flexion, hip strength, FABER; and two studies [2,8] used the Western Ontario and McMaster University (WOMAC) Osteoarthritis Index, of which one study [8] also used functional tests such as the five times sit-to-stand test and the 50-foot walk test, and the other study [2] also used functional tests, such as timed up and go test, 30 s sit to stand test, and 40 m self-paced walk test.

In Hoeksma’s study [15], a statistically significant effect of manual therapy compared with therapeutic exercise on functionality was found.

Harris Hip Score (HHS) scores at the 5-week follow-up improved by 15.3 points in the manual therapy group while improving by 4.1 points in the therapeutic exercise group. At 17 weeks, they improved by 14.4 points in the manual therapy group, while they improved by 2.9 points in the therapeutic exercise group. At 29 weeks, they improved by 16.2 points in the manual therapy group, while they improved by 6.6 points in the therapeutic exercise group.

Walking test scores (times required to walk quickly for 80 m with seven turning points inside the hospital corridor) at the 5-week follow-up improved by 8 s in the manual therapy group while improving by 0.4 s in the therapeutic exercise group. At 17 weeks, they improved by 9.5 s in the manual therapy group, while they improved by 3.3 s in the therapeutic exercise group. At 29 weeks, there were no statistically significant differences in either group. 

In Harris-Hayes’ study [6], both groups reported clinically important improvements in the HOOS function subscale and IHOT-33 scores (*p* > 0.01) at the 12-week follow-up. Furthermore, the therapeutic exercise group showed greater improvements in measurement error in hip adduction (*p* = 0.025) and pelvic drop (*p* = 0.044) during a single-leg squat and no changes in trunk tilt motion. However, no statistically significant differences were found between the two groups.

In Wright’s study [5], the differences between groups for changes in HOS ADL and Sport subscale, SANE ADL and Sport subscale, LEFS, and functional tests were not significant at the 7-week follow-up. Only the therapeutic exercise group showed clinically and statistically significant improvements in HOS ADL and Sport between baseline and the 7-week follow-up.

In French’s study [8], there were significant improvements in the treatment groups compared with the control group in WOMAC scores at 9 weeks: the therapeutic exercise group improved by 4.21 points, the combined group improved by 6.25 points, and the control group worsened by 3.18 points. There were no significant differences between the exercise group and the combined group at the 9- and 18-week follow-ups.

In Abbott’s study [2], the improvement in WOMAC compared with the usual care group was 28 points for the manual therapy (TM) plus usual care group (UC), 16 points for the exercise therapy (ET) plus usual care group, and 14 points for the TM plus ET plus UC group at the 1-year follow-up. Regardless of the joint involved (hip or knee), the greatest mean response was observed in the TM plus UC group.

In Blackman’s study [4], no statistically significant difference in LEFS scores was found between the groups analyzed at the 7-week follow-up.

#### 3.3.4. Quality of Life

Five studies [2,5,6,8,15] evaluated the effectiveness of manual therapy combined with therapeutic exercise compared with therapeutic exercise alone in quality of life. For the evaluation of this outcome, two studies [8,15] used the Short Form 36 (SF-36), and two studies [2,8] used the Patient Global Assessment (PGA), of which one study [8] also used the Hospital Anxiety and Depression Scale (HADS) and the SF-36, one study used [6] the subscale of the Hip Disability and Osteoarthritis Outcome Score (HOOS), and one study [5] used the Patient Acceptable Symptom Scale (PASS) and the Global rating of change scale (GRCS).

In Hoeksma’s study [15], no significant differences were found between the groups in the SF-36 subscales (“bodily pain”, “physical function”, “role physical function”), except for a beneficial effect of the therapeutic exercise group in the “role physical function” subscale at 5 weeks, where there was an improvement of 7.5 points in contrast to an improvement of only 3.8 points in the manual therapy group. At the 17- and 29-week follow-ups, there were no statistically significant differences in either group.

In Harris-Hayes’ study [6], both groups reported clinically important improvements in HOOS quality of life subscale scores at the 12-week follow-up: The combined group improved by 21.7 points, while the therapeutic exercise group improved by 12.1 points. After statistical calculations, however, no statistically significant differences were found between the two groups. 

In Wright’s study [5], between-group differences for changes in PASS and GRCS scores were not significant at the 7-week follow-up.

In French’s study [8], no significant between-group differences were found for the SF-36 and HADS scores at the 9- and 18-week follow-ups. At 9 weeks, however, significant improvements were observed in the treatment groups compared with the control group in PGA (ET + MT) VS Control, OR (95% CI): 11.4 (3.8 to 34.5)—ET versus Control, OR (95% CI): 12.5 (4.1 to 38). No difference was found between the therapeutic exercise group and the combined group at the 9- and 18-week follow-up.

In Abbott’s studies [2,3], there is no subdivision between hip and knee at the 1- and 2-year follow-ups; therefore, the results were not considered.

#### 3.3.5. Patient Satisfaction

Regarding the evaluation of patient satisfaction, the only study that assessed this outcome was French’s study [8]. This study showed a significant difference in the results of the patient satisfaction survey for the outcome at the 18-week follow-up. The combined group was more satisfied (3.20 points) than the therapeutic exercise group (2.80 points). Unfortunately, there were no other studies with which to compare these results.

### 3.4. Risk of Bias Assessment

**Figure 2:**
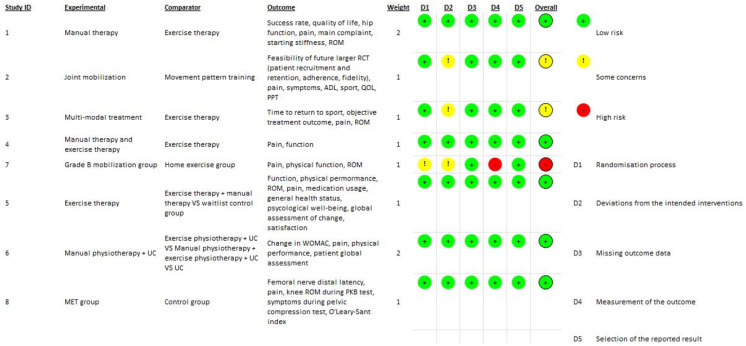
Rob-2 score evaluation.

Overall, five studies [2,5,7,8,15] rated the overall risk of bias as “low”, two studies [6,9] rated the overall risk of bias as “some concerns”, and only one study [4] rated the overall risk of bias as “high”. Overall, therefore, the methodological quality of the studies analyzed would appear to be high. The quality of the methodological evidence of the RCTs is summarized in Figure 2.

Since observational studies were not included, the NOS scale was not used.

## 4. Discussion

The aim of this systematic review was to investigate the evidence about which is the most effective treatment between manual treatment combined with therapeutic exercise and treatment based on therapeutic exercise alone for the management of hip-related nonspecific musculoskeletal diseases. 

Hence, the first element that deserves discussion regarding this systematic review is the significant difference in the type of intervention administered in the included studies [2,3,4,5,6,7,8,9,15,23]. The analysis of the individual studies shows that within the same study, the therapeutic exercise intervention differed between the comparison groups [2,6,9]. In other studies, moreover, other parallel interventions, such as paraffin therapy [9] or the administration of unspecified dosages of analgesic drugs, were added [2,4,8].

These differences within groups could have altered results because of the heterogeneity of intervention modalities. This must be regarded as a very important confounding factor that undermines the reliability of the results of the studies themselves.

Another important limitation regarding intervention is that some studies claim to subject patients to therapeutic exercise interventions, when, in fact, they only propose the administration of a single stretching exercise [7]. Instead, today’s literature emphasizes how crucial it is to plan and tailor exercise to the individual patient, avoiding “one size fits all” approaches [24].

For all the outcomes (pain, ROM, functionality, quality of life), the extreme variability of the outcome measures used was highlighted, with extreme heterogeneity of the follow-ups at which they were evaluated, which did not make it possible to combine the results quantitatively [2,3,4,5,6,7,8,9,15,23]. Therefore, it is desirable that future research studies on the comparison of therapeutic exercise and manual therapy use common follow-up times and outcome measures to be able to compare data. 

What can be seen from the analysis of the studies included in this systematic review is that the studies show a high degree of contradiction about all the outcomes analyzed. Regarding pain [2,5,6,8,9], ROM [8,9], and quality of life [2,5,6,8], most of the studies demonstrate the absence of statistically significant changes between the combined group and the therapeutic exercise alone group. Studies that evaluated the comparison between hands-on and hands-off approaches in other musculoskeletal areas confirm the same contradictory effectiveness shown in this systematic review [25,26]. For example, in the management of chronic low back pain, Ulger’s study [26] states that there was a greater reduction in pain and an improvement in functional status in favor of the manual therapy group compared with a group that performed stabilization exercises alone; on the other hand, a very recent study by Sipaviciene [25] shows that there was no statistically significant difference in pain, disability and spinal mobility between groups performing home exercise and manual therapy or supervised exercise on nonspecific chronic low back pain. This should lead the clinician to use manual therapy, combined with therapeutic exercise, without demonizing one approach over the other but drawing the positive effects on the patient from every single tool available. Manual therapy interventions have been consistently recommended for a variety of conditions [27] with minimal safety concerns and should be considered one of the therapeutic tools available for the management of hip-related nonspecific musculoskeletal diseases [28]. Modern and reconceptualized models to integrate its educational, clinical, and research models have been suggested. For example, the American Physical Therapy Association defines manual therapy as “The synergistic application of movement-oriented strategies that integrates exercise and manually applied mobilization and manipulation procedures” [29]. Accordingly, manual therapies might be considered as a specific form of touch in the context of a complex therapeutic encounter [29]. Following this contemporary understanding, there has been an expansion in the models used to understand and deliver manual therapy interventions. Modern integrated manual therapy should promote a person-centered approach, aiming at empowering the patient to re-engage with activities that they value [28,30]. Therefore, a reconceptualized framework of manual therapy should rely on the humanistic domains of safety, comfort, and efficiency [28,30]. In addition, manual therapy should be underpinned by the dimensions of communication, context, and person-centered care to foster an empowering, biopsychosocial, evidence-informed approach to care [28,30]. Results of our systematic review seem to show a superiority of the therapeutic exercise group in medium- and long-term functionality compared with the combined treatment group only for hip osteoarthritis [2,15]. Recent literature regarding hip osteoarthritis seems to be in accordance with this preliminary evidence, confirming that manual therapy does not bring any benefit in addition to therapeutic exercise in the long term on functionality in subjects with hip osteoarthritis [31]. Regarding the outcome of satisfaction, no conclusions can be drawn because of the paucity of studies [8]; therefore, more studies regarding this important clinical outcome are needed. Undoubtedly, the large spectrum of pathologies included in the present systematic review may have placed a limit on the validity of the results obtained.

In fact, some studies included in our review analyzed patients with hip osteoarthritis, which could be defined as a specific pathology, leading to a limitation for our analysis, but according to our analysis, this situation is such only when this condition is diagnosed radiographically in order to provide surgery [32]. Therefore, we are aware that in the absence of this radiological examination, hip osteoarthritis is treated through a conservative approach as one of those dozens of nonspecific causes [33,34]. This is the reason that led us to follow this direction in our review. Therefore, future studies on this topic should focus on a single specific pathology to eliminate other potential confounding factors, allowing a quantitative comparison of data. Finally, although the methodological quality of the studies assessed by the risk of bias assessment appears to be high, it must be considered that there are three pilot studies [4,5,6] and two secondary analyses (long-term follow-up) [3,23], which makes the conclusions of the systematic review weaker.

### Implications of Physiotherapy Practice 

Based on the results obtained from this review and considering the evident methodological limitations of the studies analyzed, because of the presence of confounding factors, there does not seem to be a more effective intervention between that based on manual therapy combined with therapeutic exercise and that based on therapeutic exercise alone in subjects suffering from hip related nonspecific musculoskeletal diseases. Clinicians who treat musculoskeletal disorders, therefore, should use manual therapy combined with therapeutic exercise, without demonizing one approach over the other but drawing the positive effects on the patient from every single tool available. It is not possible to give a definitive answer to the initial question; therefore, it is hoped that future research studies will provide for greater homogeneity to make the results obtained comparable, both from a qualitative and quantitative point of view and with greater importance given to the patient’s perspective and assessing the patient’s satisfaction, which plays a fundamental role in choosing the best intervention strategy.

## 5. Conclusions

There seems to be no difference in effectiveness between manual therapy combined with therapeutic exercise and therapeutic exercise alone in the treatment of individuals with hip nonspecific musculoskeletal diseases. Preliminary evidence would show that the only exception could be made in hip osteoarthritis for the medium- and long-term outcomes of function, in which the addition of manual therapy would not appear to provide any additional benefit over therapeutic exercise alone. More studies that take account of the exclusion of confounding factors and adopt a more patient-centered view are needed to draw definitive conclusions.

## Figures and Tables

**Table 1:** Inclusion and exclusion criteria.

Inclusion Criteria	Exclusion Criteria
- Adults (18–65 years old) suffering of hip related nonspecific musculoskeletal disorder	- Children, teenagers (younger than 18 years old), or elderly (older than 65 years old)
	- People suffering from specific hip pathology (e.g., fracture, hip replacement, etc.)
- Comparison between manual therapy plus therapeutic exercise versus therapeutic exercise alone	- Other types of experimental and control intervention
- Outcomes: pain, ROM, function, quality of life, satisfaction	- Outcomes different from pain, ROM, function, quality of life, satisfaction
- Primary studies (RCTs, observational studies)	- Secondary studies, protocols, or gray literature
- English and Italian languages	- Languages different from English and Italian

**Table 2:** Search strategy for Medline.

No.	Research	Results
1	(“Hip disease*” [All Fields] OR “Hip disorder*” [All Fields] OR “Hip syndrome” [All Fields] OR “Hip patholog*” [All Fields] OR “Hip pain” [All Fields] OR “Groin pain” [All Fields] OR “Adductor related groin pain” [All Fields] OR “Pubalgia” [All Fields] OR “Athletic pubalgia” [All Fields] or “Adductor injur*” [All Fields] OR “Inguinal related groin pain” [All Fields] OR “Inguinal pain” [All Fields] OR “Snapping hip” [All Fields] OR “Sport* hernia” [All Fields] OR “Pubic osteitis” [All Fields] OR “Adductor tendinopat*” [All Fields] OR “Hip osteoarthrosis” [All Fields] OR “Hip osteoarthritis” [All Fields] OR “Hip arthrosis” [All Fields] OR “Hip arthritis” [All Fields] OR “Iliopsoas related groin pain” [All Fields] OR “Hip related groin pain” [All Fields] OR “Deep gluteal syndrome” [All Fields] OR “Piriformis syndrome” [All Fields] OR “Greater trochanteric pain syndrome” [All Fields] OR “Gluteal tendinopat*” [All Fields] OR “Hip bursitis” [All Fields] OR “Femoroacetabular impingment” [All Fields])	110,884
2	(“Musculoskeletal Manipulations” [MeSH Terms] OR “Manual therap*” [All Fields] OR “Hands-on” [All Fields] OR “Hand* on” [All Fields] OR “Exercise program*” [All Fields] OR “Strength training” [All Fields] OR “Resistance training” [All Fields] OR “Aerobic training” [All Fields] OR “Conservative treatment” [All Fields] OR “Rehabilitation” [All Fields] OR “Physical therapy” [All Fields])	2,130,739
3	(“Exercise” [Mesh] OR “Exercise*” [All Fields] OR “Exercise therap*” [All Fields] OR “Training” [All Fields] OR “Physical exercise*” [All Fields] OR “Physical therap*” [All Fields] OR “Physical activit*” [All Fields] OR “Physical training” [All Fields] OR “Hand* off” [All Fields] OR “Hands-off” [All Fields])	3,771,988
4	(“Effectiveness” [All Fields] OR “Efficacy” [All Fields] OR “Success*” [All Fields] OR “Usefulness” [All Fields] OR “Pain” [Mesh] OR “Pain” [All Fields] OR “Discomfort” [All Fields] OR “Function*” [All Fields] OR “Disability Evaluation” [Mesh] OR “Disability” [All Fields] OR “Incapacity” [All Fields] OR “Quality of life” [All Fields])	16,837,911
5	(“Randomized Controlled Trials as Topic” [Mesh] OR “Random Allocation” [Mesh] OR “Randomized controlled clinical trial*” [All Fields] OR “Randomized controlled trial*” [All Fields] OR “Randomized clinical trial” [All Fields] OR “Randomized trial” [All Fields] OR “Clinical trial” [All Fields] OR “Observational Studies as Topic” [Mesh] OR “Observational stud*” [All Fields])	1,970,511
6	(“Systematic review” [All Fields] OR “Review” [All Fields] OR “Arthroplasty” [All Fields] OR “Hip replacement” [All Fields] OR “Hip arthroscopy” [All Fields] OR “Hip surgery” [All Fields])	4,336,195
7	1 AND 2 AND 3 AND 4 AND 5 NOT 6	430

**Table 3:** Characteristics of the population.

Sex (%)	60% F–40% M
Type of musculoskeletal disorder (%)	-Osteoarthritis: 60% -Groin pain: 14% -FAI: 3% -Paresthetic myalgia: 5%
Onset (%)	Chronic (>3 months): 77%

**Table 4:** Type of intervention administered.

Number of patients [2,3,4,5,6,7,8,9,15,23]	Type of intervention administered [2,3,4,5,6,7,8,9,15,23]
56	Manual therapy
54	Manual therapy in combination with usual care
160	Exercise therapy
63	Exercise therapy in combination with usual care
109	Manual therapy in combination with exercise therapy
61	Manual therapy in combination with exercise therapy and usual care
51	Usual care
43	Waiting list (no intervention)

## Data Availability

The authors confirm that the data supporting the findings of this study are available within the article and its Supplementary Materials.

## Supplementary Materials

The following supporting information can be downloaded at https://www.mdpi.com/article/10.3390/jfmk9040262/s1, Supplementary File S1: Excel sheet for data extraction.
